# Adherence to Iron-Folic Acid Among Pregnant Women Attending Antenatal Care in Southern Ethiopia, 2022

**DOI:** 10.1089/whr.2023.0020

**Published:** 2023-08-25

**Authors:** Temesgen Geta Hardido, Adisu Ashiko Mikamo, Cherinet Tilahu Legesse

**Affiliations:** ^1^ School of Nursing, College of Medicine and Health Science, Wolaita Sodo University, Wolaita, Ethiopia.; ^2^ School of Midwifery, College of Medicine and Health Science, Wolaita Sodo University, Wolaita, Ethiopia.

**Keywords:** adherence, iron, folic acid, pregnant women, Wolaita zone, public primary hospitals

## Abstract

**Background:**

Among the micronutrient deficiencies, iron and folic acid are the most common and frequently occur in pregnant women. So, the objective of this study was to assess the adherence to iron and folic acid among pregnant women booking antenatal services in the study area.

**Materials and Methods:**

A facility-based cross-sectional study design was used from January to April 2022. A structured questionnaire was used to collect data using systematic sampling techniques to approach 339 pregnant women. Data were entered and analyzed using SPSS software version 20. A descriptive analysis was performed. Adjusted odds ratios and corresponding 95% confidence intervals (CIs) were used, and statistical significance was reported at *p*-values <0.05 with 95% confidence.

**Results:**

In this study, 339 (99%) pregnant women participated. The mean age of the respondents was 28.04 years old, with a standard deviation of 5.3 years. The rate of adherence to iron and folic acid supplementation (IFAS) in pregnant women was 62.8%. Mothers under 19 years old (adjusted odds ratio [AOR] = 0.025; 95% CI [0.003–0.218]), daily (AOR = 0.127; 95% CI [0.028–0.568]), and those with a history of miscarriage (AOR = 0.276; 95% CI [0.086–0.891]) were less likely to be using IFAS. However, greater knowledge of IFAS was positively correlated with use of the supplements (AOR = 5.56; 95% CI [1.23–8.34]).

**Conclusions:**

In this study, the adherence rate with IFAS of pregnant women in the study area was 62.8%. This indicates that one in four women is not in compliance with IFAS. Appropriate counseling and health education should be provided to pregnant women to improve compliance.

## Introduction

Micronutrient deficiencies negatively impair maternal health by affecting pregnancy outcomes, as well as infant growth and development.^1^ Among these micronutrients, iron and folic acid (IFA) deficiency is the most common, affecting maternal health.^2^ Iron deficiency impairs productivity and cognition in the general population and is the most common cause of anemia during pregnancy.^1–3^ In contrast, folic acid deficiency will cause neural tube defects in the fetus and negatively affect the pregnancy outcome. Therefore, supplementation of two micronutrients for pregnant women is an important strategy to prevent the health impacts on women and their children.^3^

According to the World Health Organization (WHO), early and appropriate supplementation of folic acid (400 g) and iron (30–60 mg) during antenatal care (ANC) can reduce perinatal, neonatal, and maternal mortality by preventing low birth weight, anemia, and encephalitis.^4^ WHO estimates that between 58% and 50% of maternal deaths are related to anemia in developing and developed countries.^4^ To address these issues, the health ministries of various countries, including Ethiopia, have introduced a policy of providing IFA to pregnant women in single or in combination in tablet form.^1–5^

Providing these micronutrients to women alone cannot lead to positive results, and the effectiveness and success of this intervention always depend on women's adherence to taking iron and folic acid tablets as prescribed by medical professionals. In a medical context, adherence refers to the extent to which clients/patients appropriately follow medical information provided by health care providers. Evidence suggests that nonadherence to iron therapy can have a significant impact on the success and complementarity of national programs in different countries.^1,2,5^

Adherence to oral iron and folic acid is an important approach to improving national program implementation and preventing birth defects and pregnancy-related anemia.^6^ Adherence to iron and folic acid supplementation (IFAS) is referred to as the intake of tablets reported at each ANC visit by the clients.^6^ The overall adherence refers to a woman who contacted an ANC clinic and took IFA pills four or more days per week during the last month before the survey or for a period greater than or equal to 90 days in the third trimester.^6–8^ IFAS is the primary strategy to prevent and control the health effects associated with IFA deficiency, and its effectiveness depends on women's adequate adherence to the iron and folic acid tablets provided.^4^

The literature has shown that overall IFA adherence status to IFA varies from country to country: 38% in Pakistan,^9^ 51.14% in Iran,^10^ 51% in Senegal,^11^ 65% in Sudan,^12^ and 41.4% of pregnant women in Ethiopia adhered to IFA.^13^ This indicates that across the world there is a great challenge related to an adherence to IFA. An assessment of pregnant women's additional IFA compliance status and related factors is mandatory for all responsible agencies. In Ethiopia, the overall compliance status is still very low, below 50%, and varies from region to region.^13^ In addition, no previous studies were performed in the present study area. Therefore, the study aimed to evaluate the compliance of pregnant women who booked ANC services in the study area with the IFA.

## Methodology

### Study area, design, and period

A cross-sectional study was performed in primary public hospitals in the Wolaita zone from January to April 2022. The Wolaita zone is one of the southern ethnic regions. The zone is 356 km from Addis Ababa, the capital of Ethiopia. Based on the 2020 report from the Central Statistics Office (CSA), the population of Wolaita District is estimated at 5,385,782. Of these, 2,687,021 populations are represented by men and 2,698,261 by women. There are seven public primary hospitals (PHs) in the area, such as Tebela PH, Bodit PH, Bombe PH, Halale PH, Bitana PH, Gasuba PH, and Bale PH. A total of five hospitals were included in the study. These public hospitals provide medical services to the entire population of Wolaita and other surrounding areas.

### Population and eligibility criteria

Pregnant women who were taking IFA supplements at the time of data collection in all settings were considered as the study population. All pregnant women who attended ANC during data collection and who had at least one previous antenatal visit and received a 1-month iron and folate supplement for the current pregnancy before the date of the interview were included. However, women with mental disorders, unable to hear or speak, and women who came to ANC for the first time were excluded from the study.

### Sample size determination and its procedures

The required sample size for this study was determined using single population proportion estimation formula and considering the following assumptions; the rate of adherence to IFAS among women was taken as 71.8%,^14^ 95% of confidence interval (CI), 5% of acceptable margin of error, and 10% of no response rate. A total of five medical facilities (Bodit PH, Gasuba PH, Bombe PH, Bale PH, and Halale PH) were selected in the study. For each hospital, the average monthly patient flow in the first and second quarters of 2021 has been determined. The estimated number of client flows is 753 (Bodit PH = 207, Gasuba PH = 175, Bombe PH = 111, Bale PH = 124, and Halale PH = 136), then a pro-rated final sample size is allocated to this center's services considering their monthly customer flow. Finally, subjects were sampled using systematic random sampling (K^th^ = N/sample size, 753/342 = 2.2), so that all other customers who visited each hospital were selected as study units at each service center up to the total sample size for the study obtained.

### Operational definition

#### Adherence to IFA

IFA adherence requires that pregnant women take four IFA tablets at least once per week in the previous month of IFAS.^7^

#### Nonadherence

A pregnant women takes less than four IFA tablets per week in the previous month of IFAS.^7^

#### Good knowledge

Pregnant women who scored the mean value and above in knowledge related questions were considered to have good knowledge about IFA.^6^

#### Poor knowledge

Pregnant women who scored less than mean value on knowledge related questions were considered to have good knowledge about IFA.^6^

### Data collection and its analysis

A structured questionnaire was adapted from the previous studies,^5–7^ which consists of sociodemographic factors of the respondents, obstetric related factors, and health system related factors. Data were collected through face-to-face interviews. Based on communication skills with the client, eight BSc nurses were selected for data collection. Four health officers were recruited as supervisors. One day of training was provided to the data collector and supervisor regarding the objective, data collection tools, procedures, and interview methods that were supposed to be applied during the collection period. The selected participants were informed by the data collectors. From the selected participants, consent was obtained and the data were collected.

The questionnaire was prepared in English and then translated into the Amharic language by experts and again translated back to English to increase consistency. To maintain completeness and consistency, data collectors were closely supervised before and during the data collection process. The supervisors followed the correct implementation of the procedure and checked the completeness and logical consistency after data collection.

The completeness and consistency of the data were checked. Then, it was coded and entered onto Epi Data 3.1. For further analysis, data were exported to SPSS 25.0. Descriptive statistics of different variables were presented by frequency and percentage using tables and pie charts. A binary logistic regression test was used to compute crude odds ratio with its 95% interval to test the associations between dependent and independent variables. The variables found to be *p* < 0.25 in the bivariate analysis were taken as a candidate for multivariate analysis. Finally, Multivariate analysis with adjusted odds ratio (AOR) was used to control possible confounders and to determine predictors of adherence status to IFAS among the study participants. A *p*-value of <0.05 was considered as the criterion for statistical significance.

## Results

### Sociodemographic characteristics

A total of 339 (99%) study participants fully responded to the interview. The mean age of respondents was 28.04 years, with a standard deviation of 5.3 years, and the majority of mothers were between the ages of 25 and 39 years. Ninety-nine (29.2%) of the respondents are government employees, and 324 (95.6%) of them are married. In terms of educational attainment, 94 respondents educated up to diploma or higher. The majority of the study participants were from the Wolaita ethnic group. Regarding family size, 165 (48.7%) participants had 1 to 3 surviving children (Table 1).

**Table 1.** Sociodemographic Characteristic of the Respondents in Wolaita Zone Public Primary Hospitals, 2022 (*n* = 339)

Variables	Categories	Frequency	Percentage
Age in years	≤19	15	4.4
	20–24	55	16.2
	25–29	157	46.3
	30–34	80	23.6
	≥35	32	9.4
	Total	339	100.0
Marital status	Married	324	95.6
	Single	7	2.1
	Others^a^	8	2.4
Monthly income	≤3,934	204	60.2
	3,935–9,055	129	38.1
	≥9,056	6	1.8
Educational status of mothers	No formal education	23	6.8
	Primary (1–8)	72	21.2
	Secondary (9–12)	150	44.2
	Diploma and above	94	27.7
Occupation status of mothers	Gov't employee	99	29.2
	Non-governmental	12	3.5
	Self-employed	53	15.6
	Merchant	70	20.6
	House wife	75	22.1
	Others^b^	30	8.8
Husband education	No formal education	22	6.5
	Primary (1–8)	43	12.7
	Secondary (9–12)	137	40.4
	Diploma and above	137	40.4
Husband occupation	Gov't employee	124	36.6
	NGO employee	13	3.8
	Self-employed	34	10.0
	Merchant	113	33.3
	Others^b^	55	16.2
Religion	Protestant	162	47.8
	Orthodox	125	36.9
	Muslim	36	10.6
	Catholic	16	4.7
Ethnicity	Wolaita	177	52.2
	Oromo	42	12.4
	Kembata	53	15.6
	Amara	39	11.5
	Others^c^	28	8.3
Family size	1–3	165	48.7
	4 and above	174	51.3

^a^ Divorced, widowed.

^b^ Student, daily laborer, car driver farmer.

^c^ Sidama, gurage, hadiya.

### Obstetric-related factors of the respondents

In this study, 132 (38.9%) of study participants were multiparas. The majority (61.1%) of them attended ANC 2 times and 263 (77.6%) of study participants had planned last pregnancy. Of the participants, 62.8% who were using IFAS had skipped some doses. The main reasons for skipping doses were gastritis (93, 43.1%) and forgetfulness (73, 34.3%) (Table 2).

**Table 2.** Obstetric Health Related Factors Among Respondents in Wolaita Zone Public Primary Hospitals, 2022 (*n* = 339)

Variables	Categories	Frequency	Percentage
Current visit trimester	Second trimester	168	49.6
	Third trimester	171	50.4
Planned pregnancy	No	76	22.4
	Yes	263	77.6
Previous place of ANC follow-up	Hospital	259	76.4
	Health center	71	20.9
	Private clinic	9	2.7
The first registration for ANC	≤4 months	265	78.2
	≥5 months	74	21.8
Number of ANC visits	Two times	207	61.1
	Three times	96	28.3
	≥4 times	36	10.6
Skipped IFAS	No	126	37.2
	Yes	213	62.8
Reason of skip	Forgetfulness	73	34.3
	Travel	9	4.2
	Constipation	16	7.5
	Gastritis	93	43.7
	Vomiting	16	4.7
	Absence in health facility	6	2.8
Gravidity	Primi-gravida	99	29.2
	Multigravida	240	70.8
Parity	Nulliparous	105	31.0
	Primiparas	102	30.1
	Multipara	132	38.9
History of abortion	No	280	82.6
	Yes	59	17.4
History of still birth	No	329	97.1
	Yes	10	2.9

ANC, antenatal care; IFAS, iron and folic acid supplementation.

### Health system related factors

More than half (56.3%) of women took 30 minutes or more to get to a health facility, and 249 (73.5%) of the respondents spent 30 minutes or more to wait to be used in service. Two hundred ninety (64.6%) of the respondents were counseled about the benefits of IFAS. More than half (55.2%) were not informed about the frequency of IFAS use. Similarly, 212 (62.5%) women were not informed about the duration of IFAS use. Two hundred ten (61.9%) of respondents were not informed of possible side effects. More than half (62.8%) of women were not counseled on how to manage side effects. Just over half (57.8%) of women were counseled about anemia (Table 3).

**Table 3.** Health System Related Conditions of Respondents in Wolaita Zone Public Primary Hospitals, 2022 (*n* = 339)

Variables	Categories	Frequency	Percentage
Facility distance	<30 minute	148	43.7
	≥30 minute	191	56.3
Waiting time	<30 minute	90	26.5
	≥30 minute	249	73.5
Encountered with shortage of IFA	No	262	77.3
	Yes	77	22.7
Counseled on benefit of IFAS	No	120	35.4
	Yes	219	64.6
Counseled how often to take IFAS	No	187	55.2
	Yes	152	44.8
Counseled how long to take IFAS	No	212	62.5
	Yes	127	37.5
Counseled possible side effects	No	210	61.9
	Yes	129	38.1
Counseled on managing side effects	No	213	62.8
	Yes	126	37.2
Counseled about anemia	No	143	42.2
	Yes	196	57.8

### Respondents' knowledge on IFAS

**FIG. 1.**
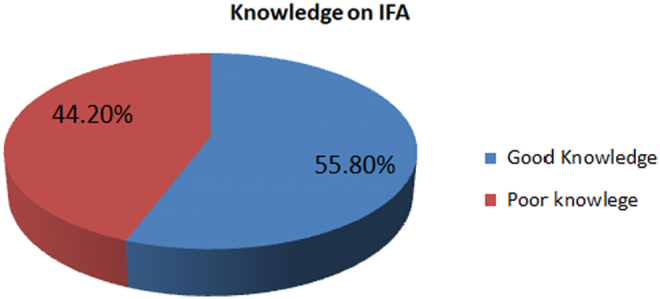
Knowledge on IFAS of the respondents. IFAS, iron and folic acid supplementation.

In more than half, 189 (55.8%) had good knowledge on anemia and IFAS and 150 (44.2%) had poor knowledge on anemia and IFAS (Fig. 1).

### Prevalence of adherence to IFAS

From a total of 339 participants included in this study, 62.8% of the participants adhered to iron-folic acid supplementation and 37.2% of them nonadhered to IFA (Table 2).

### Factors associated with IFA adherence

Significant predictors of folic acid supplementation adherence included age, maternal occupation, knowledge of IFA, and a history of abortion. Persons under 19 years of age were much less likely to be using IFAS than those in the reference group of women 35 years or older (AOR = 0.025; 95% CI [0.003–0.218]), *p* < 0.001, but otherwise use of IFAS was similar across age groups. Women who are day laborers are 87.3% less likely to use IFA than those working for the government (AOR = 0.13; 95% CI [0.028–0.568]; *p* = 0.007); however, there was nonstatistical difference in adherence among the other occupations. Women with a history of miscarriage were 73% less likely to comply with IFAS than women with no history (AOR = 0.276; 95% CI [0.086–0.891]; *p* = 0.031). Finally, women's knowledge of IFA was positively associated with IFAS. Mothers with good knowledge of IFAS were 5.56 times more likely to adhere to IFAS than mothers with poor knowledge (AOR = 5.56, 95% CI [1.23–8.34]; *p* = 0.020) (Table 4).

**Table 4.** Factors Associated with Iron and Folic Acid Adherence in Wolaita Zone Public Primary Hospitals, 2022 (*n* = 339)

Variables	Categories	COR	AOR	***p***
Age in years	≤19	0.028 (0.005, 0.167)	0.025 (0.003, 0.218)^a^	0.001
	20–24	0.278 (0.093, 0.831)	0.745 (0.193, 2.876)	0.670
	25–29	0.461 (0.167, 1.272)	0.986 (0.292, 3.331)	0.982
	30–34	0.459 (0.157, 1.338)	0.384 (0.108, 1.369)	0.140
	≥35	1	1	
Occupation status of mothers	Gov't employee	1	1	
	Non-gov't	1.068 (0.268, 4.252)	2.205 (0.364, 13.360)	0.390
	Self-employee	0.502 (0.248, 1.017)	0.984 (0.413, 2.344)	0.971
	Merchant	0.956 (0.479, 1.908)	1.507 (0.635, 3.577)	0.352
	House wife	0.671 (0.349, 1.290)	0.631 (0.268, 1.485)	0.291
	Daily laborer	0.277 (0.094, 0.819)	0.127 (0.028, 0.568)^a^	0.007
	Student	1.306 (0.338, 5.052)	6.462 (0.755, 55.282)	0.088
History of abortion	No	0.282 (0.129, 0.619)	0.276 (0.086, 0.891)^a^	0.031
	Yes	1	1	
Knowledge on IFA	Good	3.3 (0.89–7.1)	5.56 (1.23–8.34)	0.020
	Poor	1	1	

^a^ Statistically significant association in multivariate analysis, “1” reference group.

AOR, adjusted odds ratio; COR, crude odds ratio.

## Discussion

The results of this study showed that the overall IFA membership rate of the study participants in the study area was 62.8%. This finding is consistent with studies from different regions of Ethiopia, such as Dire Dawa (71.8%),^14^ Tikur anbessa (63.6%),^15^ and Sidama (64.6%).^16^ Possible reasons for this agreement could be similarities in socioeconomic status, background, and similar study design. However, this result is lower compared with studies conducted in Mozambique (79%)^6^ and northern Ethiopia (76.9%).^17^ This difference could be due to geographic location, study period, sample size, and women's perceptions.

Being a day worker negatively and significantly affects IFA membership. This study found that day workers were 87.3% less likely to comply with an IFA supplement than the other group. The reason may be that daily workers have little access to information; most of them have low education levels and low economic conditions, making it difficult to access medical facilities.

It is well known that adherence of IFAS positively and significantly associated with women's educational level.^13,18–20^ Mothers with good knowledge of IFA were 5.56 times more likely to comply with IFA than mothers who had poor knowledge. This may involve a mother who has adequate knowledge and is able to follow a health care provider's recommendation to comply with IFAS.

Previous miscarriage history was identified as an explanatory variable significantly associated with adherence to IFAS. Compliance with IFAS was 73% lower in study participants with a history of miscarriage than in their opposite group. This finding is supported by a systematic review conducted in Ethiopia.^19^ This may be because pregnant women with a history of abortion may have a strong perception of fear that the abortion might recur. As a result, this may therefore prevent them from emphasizing their ANC and additional IFA compliance.

In the sample of respondents, age was significantly associated with adherence to the IFA.^21^ Pregnant women under the age of 19 were 99.5% less likely to adhere with IFAS than those over the age of 35. This finding was supported by study findings in India^5^ and Pakistan.^9^ The reason may be due to the increasing age of pregnant mothers, as more mothers have antenatal visits per pregnancy, which should raise awareness of IFAS. The study's limitations include: (1) a cross-sectional study, which cannot assess cause–effect effect; (2) IFA adherence determined by pregnant women's self-report may underestimate the prevalence of nonadherence compared with objective measures like pill count and biological assay medication adherence measures; (3) some women who do not receive any prenatal care and are unlikely to receive IFAS are not included in this study and that attention to increasing the number of women who attend prenatal clinics is important; and (4) the possibility of recall bias.

## Conclusions

In this study, 62.8% of pregnant women in the study area adhered to IFAS. Age, the mother's profession, her knowledge of folic acid and iron, as well as her history of abortion, were linked to her compliance with iron supplementation. In addition, 22.7% of women stated that their area may have a shortage of IFA. Therefore, to improve pregnant women's adherence, we recommend that they get proper counseling and health education. The top hospital administrators and health care professionals should concentrate on the identified factors as well. IFAS should be a priority for all stakeholders, including the Federal Ministry of Health, Regional Health Bureau, and Zonal Health Department. To support IFAS, it is necessary to improve community-based supplements, counseling, and prenatal care accessibility and quality.

## Data Availability

The datasets generated and/or analyzed during the current study are not publicly available to prevent any kinds of misuse by the public before publication but are available from the corresponding author upon reasonable request.

## Abbreviations Used

ANC: antenatal care
AOR: adjusted odds ratio
CI: confidence interval
COR: crude odds ratio
IFA: iron and folic acid
IFAS: iron and folic acid supplementation
PH: primary hospital
WHO: World Health Organization
